# Accommodating multiple potential normalizations in microbiome associations studies

**DOI:** 10.1186/s12859-023-05147-w

**Published:** 2023-01-19

**Authors:** Hoseung Song, Wodan Ling, Ni Zhao, Anna M. Plantinga, Courtney A. Broedlow, Nichole R. Klatt, Tiffany Hensley-McBain, Michael C. Wu

**Affiliations:** 1 Public Health Sciences Division, Fred Hutchinson Cancer Center, Seattle, WA USA; 2 Department of Biostatistics, Johns Hopkins Bloomberg School of Public Health, Baltimore, MD USA; 3 Department of Mathematics and Statistics, Williams College, Williamstown, MA USA; 4 Division of Surgical Outcomes and Precision Medicine Research, Department of Surgery, University of Minnesota School of Medicine, Minneapolis, MN USA; 5 McLaughlin Research Institute for Biomedical Sciences, Great Falls, MT USA

**Keywords:** Normalization, Different library size, Omnibus approach, Cauchy combination test

## Abstract

**Background:**

Microbial communities are known to be closely related to many diseases, such as obesity and HIV, and it is of interest to identify differentially abundant microbial species between two or more environments. Since the abundances or counts of microbial species usually have different scales and suffer from zero-inflation or over-dispersion, normalization is a critical step before conducting differential abundance analysis. Several normalization approaches have been proposed, but it is difficult to optimize the characterization of the true relationship between taxa and interesting outcomes.

**Results:**

To avoid the challenge of picking an optimal normalization and accommodate the advantages of several normalization strategies, we propose an omnibus approach. Our approach is based on a Cauchy combination test, which is flexible and powerful by aggregating individual *p* values. We also consider a truncated test statistic to prevent substantial power loss. We experiment with a basic linear regression model as well as recently proposed powerful association tests for microbiome data and compare the performance of the omnibus approach with individual normalization approaches. Experimental results show that, regardless of simulation settings, the new approach exhibits power that is close to the best normalization strategy, while controling the type I error well.

**Conclusions:**

The proposed omnibus test releases researchers from choosing among various normalization methods and it is an aggregated method that provides the powerful result to the underlying optimal normalization, which requires tedious trial and error. While the power may not exceed the best normalization, it is always much better than using a poor choice of normalization.

**Supplementary Information:**

The online version contains supplementary material available at 10.1186/s12859-023-05147-w.

## Introduction and motivation

Microbial communities have been revealed to be closely related to many conditions, such as obesity [1–3], diabetes [4–6], and HIV [7–9]. With the development of high-throughput sequencing technologies enabling large-scale microbiome studies, human microbiome profiling studies for health conditions and diseases are gaining more attention. A central objective of human microbiome profiling studies is to identify individual bacterial taxa related to host outcomes, exposures, or other variables of interest among the meta data. This provides an important understanding of the mechanisms underlying different outcomes as well as host responses to exposures and potential contribution to therapeutic interventions.

The most common approach for identifying individual taxa related to variables of interest is to test the association between the variable and the abundance of each taxon, one by one. Many differential abundance analysis approaches have been proposed based on the linear model [10, 11], phylogenetic tree [12, 13], and zero-inflated model [14–16]. Based on these tests, the *p* value for the association of each taxon is generated and statistical significance is determined after controlling for multiple testing. On the other hand, global association tests have also been proposed to accommodate general dependency patterns between overall microbiome composition and profiles of other types of genomic data, including kernel-based tests [17–20] and distance-based tests [21–23].

Unfortunately, intrinsic challenges of microbiome data often make it difficult to identify differentially abundant taxa. For example, a central challenge of microbiome profiling studies is the issue of differential library size (total counts per sample), which is difficult to control [24] and does not reflect actual differences in microbial communities. Under-sampling in some individuals may also exacerbate zero-inflation and over-dispersion, leading to substantial power loss [25].

Normalization, broadly defined, is a strategy for overcoming differences in library size [26]. Failure to harmonize library sizes can lead to a severe loss of power as the scale of assessed abundances is essentially different for each sample. Common approaches to normalization include scaling the observations to have a unit sum (such that the norm of the abundances for each sample is equal to 1—the original definition of normalization), and scaling by other measures of central tendency, among others (see [27]). Theoretically, as long as read depth is not systematically confounded with the variable of interest, analysis under any normalizations is valid. However, in addition to philosophical differences between different normalization approaches, different normalizations also implicitly specify the expected relationship between each taxon under consideration and the outcome. A normalization that results in a better characterization of the true relationship between taxa and outcomes will lead to better power. Yet, the best normalization (leading to the highest power) is difficult to ascertain, as this depends on the unknown true state of nature and may also be different depending on the taxon under consideration.

To address the challenge of picking a single, optimal normalization, we develop an omnibus approach wherein we consider analyzing the data under several different normalization strategies. We then aggregate the results from different normalization approaches while adjusting for the fact that different normalizations have been used, in order to prevent *p*-hacking. Our approach is based on a Cauchy Combination Test (CCT) [28] which allows aggregation of correlated *p* values (calculated under different normalizations). Simulations show that our approach often leads to power similar to, or exceeds, the best individual normalization strategy, while still protecting the false discovery rate.

## Methods

We assume a study in which there are *n* samples on which *p* taxa are measured. Let the raw vector of abundances for the $i^{th}$ sample be $X_i$. For a given normalization $\mathcal {T}(\cdot )$, we set $\tilde{X}_i = \mathcal {T}(X_i)$ and $\tilde{X} = \mathcal {T}(X) = [\mathcal {T}(X_1),\ldots , \mathcal {T}(X_n)]$. We further assume that there are *J* different normalizations that can be considered such that we have $\mathcal {T}_1(\cdot ), \mathcal {T}_2(\cdot ), \ldots , \mathcal {T}_J(\cdot )$. We focus on the objective of identifying individual bacterial taxa as associated with the outcome of interest.

The fundamental challenge that we hope to address is that it is unclear which normalization to use. Different normalizations correspond to different interpretations and implications of different bacterial taxa. Consequently, we propose a strategy in which we consider multiple potential normalizations. In this section, we first describe different, commonly used normalization strategies before outlining the specific approach for implicating taxa across different normalization approaches. We further describe simulation settings for evaluating the power and type I error of our strategy.

### Common normalization approaches

Normalization is an important step for reducing variability in the data due to differential library size. Some easily applicable normalization strategies include:

(i) **None** $$\mathcal {T}(X_i) = X_i$$.
(ii) **Rarefaction** Each sample’s observed counts are sub-sampled such that the total count is the same for all samples.
(iii) **Total sum scaling (TSS)** Observed counts are scaled by the sample’s library size (sum of counts).
(iv) **Cumulative sum scaling (CSS)** Observed counts are scaled by the sum of counts up to a cutoff quantile^1^. (see details in [29]).
(v) **Center Log Ratio (CLR) transform** Observed counts are divided by the geometric mean of the sample’s counts, then log-transformed.

Even though these normalization strategies work well under many settings, some concerns are discussed in the literature: artificial uncertainty in the sub-sampling step (rarefaction) [30], a bias in differential abundance estimates (TSS) [31], and uncertainty in the selection of quantile (CSS). Hence, it is difficult to find an optimal normalization approach and it depends on the unknown form of relationship between microbiome data and interesting outcomes.

Given the inherent challenge of selecting a single optimal normalization strategy, we propose to simply apply multiple normalization approaches. Specifically, for each choice of normalization, we apply a valid statistical test to get the *p* value for the association between each taxon and the variable of interest. In the next section, we describe how we combine the *p* values to get a single omnibus *p* value for each taxon.

### Combining results across normalizations via the cauchy combination test

Assume that after applying each normalization, we apply a valid test to assess the association between each taxon and the variable of interest such that we have a $J\times p$ matrix of individual *p* values ${\textbf {P}}$ with $p_{j,k}$, the *p* value for the $k^{th}$ taxon after applying the $j^{th}$ normalization strategy to the dataset.

Given ${\textbf {P}}$, we apply the Cauchy combination test (CCT) [28] for each taxon to obtain the omnibus *p* value. It is well-known that the Cauchy combination test is useful for dealing with sparse alternatives, high-dimensional large-scale datasets, and small *p* values, which are common situations in GWAS. In particular, the analytic *p* value approximation by the Cauchy distribution is very accurate under arbitrary dependency structures. Hence, in practice, the test only requires the individual *p* values as input, so this omnibus testing procedure is very fast. Specifically, for $k = 1, \ldots , p$, we set

1 $$\begin{aligned} p_k = \frac{1}{J} \sum _{j=1}^J \tan \{(0.5-p_{j,k})\pi \} \end{aligned}$$

to be the final *p* value for the *k*th taxon and $p_{k}$ incorporates each normalization strategy.

Though CCT is convenient and exact for any number of *p* values, it suffers the drawback of sensitivity to *p* values at or near 1. Specifically, the Cauchy combination *p* value, $p_{k}$, converges to 1 as one of $p_{j,k}$ approaches 1 $(j = 1, \ldots , J)$. This can happen for tests of discrete data or when the model to derive *p* values is mis-specified. To address this, we propose to use a truncated Cauchy combination test proposed by [32]:

2 $$\begin{aligned} p_k = \frac{1}{J} \sum _{j=1}^J \tan \{(0.5-\min (p_{j,k},1-\epsilon ))\pi \}, \end{aligned}$$

where $\epsilon = 0.01$. This prevents the overshoot of $p_{k}$ over the threshold $1-\epsilon$.

### Simulation setup

To check the performance of the method, we first follow the simulation setup in [16] using data generated to mimic a real data set. Specifically, we simulate data from the Coronary Artery Risk Development in Young Adults Study (CARDIA) [33] which aimed to investigate microbial taxa related to cardiovascular disease risk factors. The broader parent study [34] was balanced in terms of race (black or white), education (more than high school or not), and age. Between 1987 and 2016, each subject had up to eight follow-up visits. A variety of cardiovascular disease-related parameters, as well as physical measurements and lifestyle factors, were gathered.

We follow the preprocessing of [16] and focus on microbiome count data aggregated at the genus-level. The processed data contains data on 106 genera for 549 subjects. Our goal is to identify differentially abundant taxa between subjects with high blood pressure (HBP) and without HBP and examine how powerful the omnibus approach is over individual normalization strategies. Here, we treat blood pressure as a binary variable (HBP vs. non-HBP). We then consider two scenarios based on unadjustment/adjustment for covariates:

- Setting 1 (type I error and power of individual-level analysis on a single taxon): We select ‘Streptococcus’ taxon, which is differentially abundant with strong differences in the mean abundance by HBP status, and test the association between the selected taxon and HBP without adjustment for other covariates. To assess the type I error rate, we generate 600 samples from the empirical distribution functions (edf) of the normalized abundance in subjects without HBP. To assess the power of the test, we generate 300 samples each from the edf of HBP and non-HBP groups. We also examine cases where the two groups are mixed by $\delta$% with each other. We simulate 10,000 datasets and the significance level is set to be 0.01.
- Setting 2 (type I error and power of individual-level analysis on an OTU table): We create the starting dataset by rarefying the CARDIA dataset 10 times and averaging the resulting datasets. With this starting data, we fit each of the genera by the two-part quantile regression model (see Additional file 1: Figure S1):
  $$\begin{aligned} \text {logit}\{P(D=1|X)\}&= \gamma _{0}+\gamma _{1}\text {HBP}+\gamma _{2}\text {age}+\gamma _{3}\text {physical activity} \\&\ \ \ \ +\gamma _{4}\text {diet quality score}, \\ Q_{Y}(\tau |X,Y>0)&= \beta _{0}(\tau )+\beta _{1}(\tau )\text {HBP}+\beta _{2}(\tau )\text {age}+\beta _{3}(\tau )\text {physical activity} \\&\ \ \ \ +\beta _{4}(\tau )\text {diet quality score}, \end{aligned}$$
  where $D = I(Y>0)$ is a binary indicator of the presence of genus, and $\gamma _{i}$’s and $\beta _{i}$’s are estimated by the starting data and non-zero observations of the starting data using $\tau = 0.01,\ldots ,0.99$, respectively. To assess the type I error rate, we generate *n* samples by resampling each of the real covariates with replacement independently and simulate *D* with the constraint $\gamma _{1}=\beta _{1}(\tau )=0$. If $D=0$, we assign 0 as the count. If $D=1$, we simulate the count by the inverse CDF method: compute $Y=\beta _{0}(u)+\beta _{2}(u)\text {age}+\beta _{3}(u)\text {physical activity}+\beta _{4}(u)\text {diet quality score}$, where $u\sim U(0,1)$ and round it to the nearest integer. To assess the power of the test, we follow the same procedure without the constraint. We simulate 1000 datasets and the significance level is set to be 0.05.

For each test, normalization strategies (i)–(v) are considered and we compare the omnibus *p* value with the *p* values based on each normalization strategy. To obtain the *p* values, we apply the simple linear regression, a zero-inflated quantile approach (ZINQ) proposed by [16], a quantile regression using a rank score function and ignoring zero inflation (QRank) proposed by [35]. In addition, the mixing rate $(\delta )$ and sample size (*n*) are chosen so that the results can be compared well between normalization strategies.

We also consider the simulation setup in [36] to examine the performance of the omnibus method by a global microbiome association test. Specifically, we generate *n*/2 genotype data of haplotypes from African and European ancestry by randomly pairing 2 haplotypes, respectively, over a 1 MB chromosome according to coalescent theory using the cosi2 program [37]. We also generate *n* samples of microbiome OTU counts from the Dirichlet-multinomial distribution. We first estimate the parameters of the Dirichlet-multinomial distribution using a real upper-respiratory-tract microbiome dataset [38]. This dataset is publicly available by an $\texttt {R}$ package $\texttt {GUniFrac}$. This consists of 856 OTUs.

- Setting 3 (type I error and power of community-level analysis on an OTU table): To assess the type I error rate, we use the above setting without introducing any genetic effect on the microbiome. To assess the power of the test, we introduce the association between genetics and microbiome. For each individual $i = 1, \ldots , n$, let $g_{i}$ be the genotype at a chosen common SNP (with MAF $\ge$ 0.05). Then we increase the counts of the $\eta$th–20th most common OTUs by a factor $f_{i} = 1 + 1.7*g_{i}$.

For each test, we use a kernel RV coefficient (KRV) test to evaluate the overall association between genetic expression and microbiome composition. [39, 40]. We use the Bray-Curtis kernel and choose normalization strategies (i)–(iv) since CLR transformation provides negative results and this does not allow the KRV test. We simulate 1000 datasets and the significance level is set to be 0.05.

## Results

### Type I error

Table 1, 2, and 3 show the empirical size of the tests under Setting 1, 2, and 3, respectively. We see that the omnibus approach in general controls the type I error rate well, compared to other normalization approaches.

**Table 1:** Empirical size of the tests under Setting 1 at 0.01 significance level

	None	Rarefaction	TSS	CSS	CLR	Omnibus
Linear regression	0.009	0.010	0.010	0.010	0.010	0.010
ZINQ	0.010	0.009	0.010	0.010	0.012	0.010
QRank	0.009	0.010	0.009	0.009	0.009	0.009

**Table 2:** Empirical size of the tests under Setting 2 at 0.05 significance level, where *n* is the sample size

		None	Rarefaction	TSS	CSS	CLR	Omnibus
$n=700$	Linear Regression	0.048	0.048	0.049	0.050	0.050	0.051
	ZINQ	0.052	0.051	0.052	0.052	0.055	0.056
	QRank	0.050	0.050	0.050	0.049	0.049	0.052
$n=600$	Linear Regression	0.049	0.049	0.048	0.050	0.050	0.052
	ZINQ	0.052	0.052	0.055	0.053	0.055	0.056
	QRank	0.052	0.052	0.052	0.050	0.049	0.055
$n=500$	Linear Regression	0.047	0.047	0.048	0.050	0.049	0.051
	ZINQ	0.052	0.052	0.054	0.053	0.055	0.056
	QRank	0.050	0.050	0.050	0.049	0.049	0.055
$n=400$	Linear Regression	0.047	0.047	0.047	0.050	0.049	0.050
	ZINQ	0.052	0.053	0.054	0.054	0.055	0.056
	QRank	0.051	0.050	0.050	0.050	0.049	0.052

**Table 3:** Empirical size of the tests under Setting 3 at 0.05 significance level, where *n* is the sample size

	None	Rarefaction	TSS	CSS	Omnibus
$n=500$	0.049	0.049	0.049	0.049	0.046
$n=400$	0.056	0.056	0.056	0.053	0.056
$n=300$	0.058	0.058	0.058	0.053	0.056

### Power

**Fig. 1:**
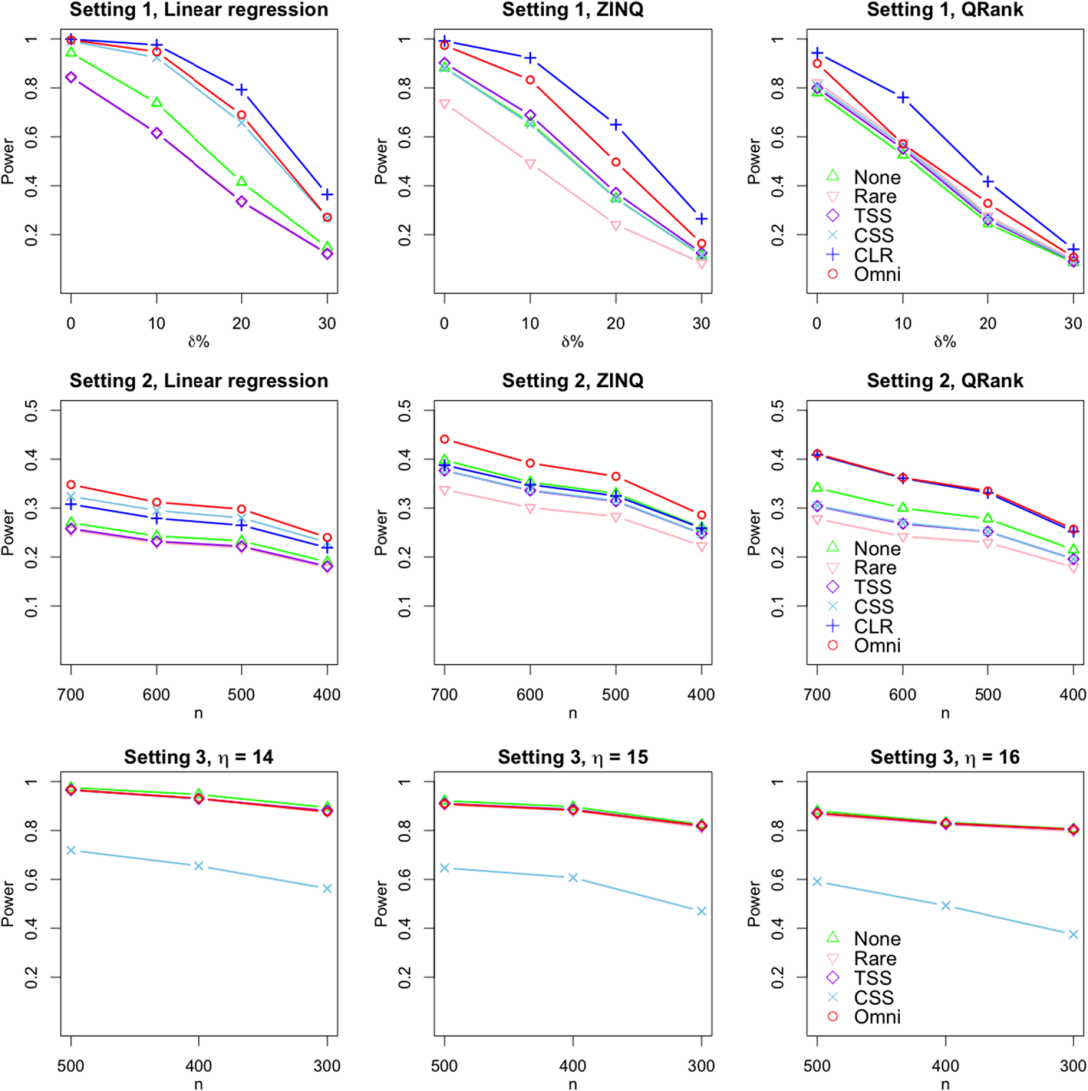
Estimated power of the tests under Setting 1, 2, and 3

Table 4, 5, and 6 show the estimated power of the tests under Setting 1, 2, and 3, respectively. Figure 1 shows their visualization. Under Setting 1, we see that CLR normalization exhibits the best performance, while rarefaction shows the worst performance. Within this gap, the omnibus approach exhibits power that is almost as high as the performance when using CLR normalization. On the other hand, under Setting 2, when applying the linear regression, CSS normalization exhibits high power, while the rarefaction approach shows the worst performance. Surprisingly, the omnibus approach exhibits the best performance, even over the CSS approach. When using ZINQ, rarefaction approach still shows the worst performance, while ZINQ achieves high power without normalization. The omnibus approach shows the best performance, the same as the linear regression case. When applying QRank, CLR approach exhibits high power, but the omnibus approach achieves the best power. Under Setting 3, the omnibus approach exhibits high power, while raw data and TSS normalization show good performance as well.

As shown in Table 4, 5, and 6, the best normalization strategy depends on different differential abundance methods and situations, and it is difficult to choose the optimal normalization strategy. However, the omnibus method generally performs well without prior or true relationship knowledge between taxa and outcomes, so this would be more efficient and useful when applying differential abundance tests in microbiome studies.

**Table 4:** Estimated power of the tests under Setting 1, where $\delta$ is the mixing proportion

		None	Rarefaction	TSS	CSS	CLR	Omnibus
$\delta =0\%$	Linear Regression	0.943	0.842	0.844	0.991	**0.999**	**0.996**
	ZINQ	0.882	0.738	0.902	0.882	**0.992**	**0.974**
	QRank	0.780	0.821	0.799	0.809	**0.943**	**0.900**
$\delta =10\%$	Linear Regression	0.738	0.613	0.616	0.924	**0.976**	**0.948**
	ZINQ	0.658	0.493	0.689	0.651	**0.923**	**0.833**
	QRank	0.526	0.576	0.551	0.564	**0.761**	**0.571**
$\delta =20\%$	Linear Regression	0.415	0.332	0.336	0.658	**0.793**	**0.690**
	ZINQ	0.348	0.241	0.371	0.346	**0.650**	**0.497**
	QRank	0.245	0.276	0.261	0.267	**0.417**	**0.328**
$\delta =30\%$	Linear Regression	0.148	0.120	0.122	0.268	**0.364**	**0.271**
	ZINQ	0.114	0.084	0.125	0.117	**0.265**	**0.164**
	QRank	0.086	0.098	0.090	0.092	**0.140**	**0.108**

The two highest powers are in bold

**Table 5:** Estimated power of the tests under Setting 2, where *n* is the sample size

		None	Rarefaction	TSS	CSS	CLR	Omnibus
$n=700$	Linear Regression	0.270	0.255	0.258	**0.324**	0.308	**0.348**
	ZINQ	**0.398**	0.338	0.377	0.377	0.388	**0.441**
	QRank	0.341	0.278	0.304	0.305	**0.409**	**0.411**
$n=600$	Linear Regression	0.243	0.229	0.232	**0.295**	0.279	**0.312**
	ZINQ	**0.353**	0.301	0.336	0.337	0.348	**0.392**
	QRank	0.300	0.242	0.268	0.270	**0.361**	**0.362**
$n=500$	Linear Regression	0.233	0.219	0.222	**0.280**	0.265	**0.298**
	ZINQ	**0.330**	0.283	0.314	0.315	0.325	**0.365**
	QRank	0.278	0.230	0.252	0.252	**0.331**	**0.335**
$n=400$	Linear Regression	0.190	0.178	0.181	**0.230**	0.219	**0.240**
	ZINQ	**0.260**	0.223	0.248	0.248	0.259	**0.286**
	QRank	0.215	0.179	0.196	0.196	**0.252**	**0.257**

The two highest powers are in bold

**Table 6:** Estimated power of the tests under Setting 3, where *n* is the sample size and $\eta$ indicates the order of taxon

		None	Rarefaction	TSS	CSS	Omnibus
$n=500$	$\eta = 14$	**0.975**	0.965	**0.966**	0.719	**0.966**
	$\eta = 15$	**0.921**	0.905	**0.910**	0.647	0.909
	$\eta = 16$	**0.880**	0.864	**0.871**	0.591	**0.871**
$n=400$	$\eta = 14$	**0.947**	0.929	0.930	0.655	**0.931**
	$\eta = 15$	**0.897**	0.881	**0.886**	0.607	0.883
	$\eta = 16$	**0.833**	0.824	0.828	0.493	**0.830**
$n=300$	$\eta = 14$	**0.894**	0.875	**0.881**	0.563	0.876
	$\eta = 15$	**0.824**	0.812	0.819	0.470	**0.820**
	$\eta = 16$	**0.806**	0.797	**0.804**	0.375	**0.804**

The two highest powers are in bold

### Real data application

In this section, we illustrate the omnibus approach on the HIV dataset analyzed in [41]. The HIV dataset is obtained by a multicolor flow cytometry-based method that separates neutrophils from other leukocytes in order to get a more precise measurement of neutrophil frequencies in the Gastrointestinal (GI) during HIV infection. This allows the identification of neurophils in blood and fresh GI issues and the calculation of the frequency of neutrophils as a percentage of all live CD45$+$ cells.

As a result, this dataset consists of colorectal biopsies from a total of 40 HIV-infected, antiretroviral therapy (ART) suppressed individuals and 35 HIV-uninfected individuals with relevant participant demographic information, such as age, sex, sexual orientation, and race/ethnicity. The authors characterized the intestinal microbiome of colorectal biopsies using 16S rRNA sequencing and studied the association between the mucosal microbiome composition of colorectal biopsies and some relevant factors. For example, based on the fact that men who have sex with men (MSM) have an increased abundance of Prevotella, independent of HIV status, and this may result in the dysbiosis previously attributed to HIV infection, the authors observed the significant association between the overall microbial composition at the genus level and sexual orientation (MSM or non-MSM). They also showed that this association remained when adjusted for age, race, and HIV status.

We utilize this dataset to illustrate how the omnibus approach accommodates several normalization strategies. Specifically, we conduct association tests between the mucosal microbiome composition of colorectal biopsies and the sexual orientation. According to the results in [41], we test the association with the sexual orientation without adjustment for other covariates or with adjustment for age, race, and HIV status. Here, we fix the false discovery rate (FDR) at 20%.

**Table 7:** Number of differentially abundant taxa based on the sexual orientation

		None	Rarefaction	TSS	CSS	CLR	Omnibus
without covariates	Linear Regression	1	5	7	**24**	**26**	21
	ZINQ	13	11	8	16	**24**	**17**
	QRank	10	9	13	**20**	10	**15**
with covariates	Linear Regression	1	6	6	**20**	**23**	19
	ZINQ	0	0	11	1	**24**	**16**
	QRank	**13**	7	6	5	6	**8**

The two highest powers are in bold

Table 7 shows the number of differentially abundant taxa out of 108 taxa based on the sexual orientation at 20% FDR. We see that the best normalization approach is different for each case, but the omnibus approach in general identifies nearly as many significant taxa as the best normalization approach.

**Fig. 2:**
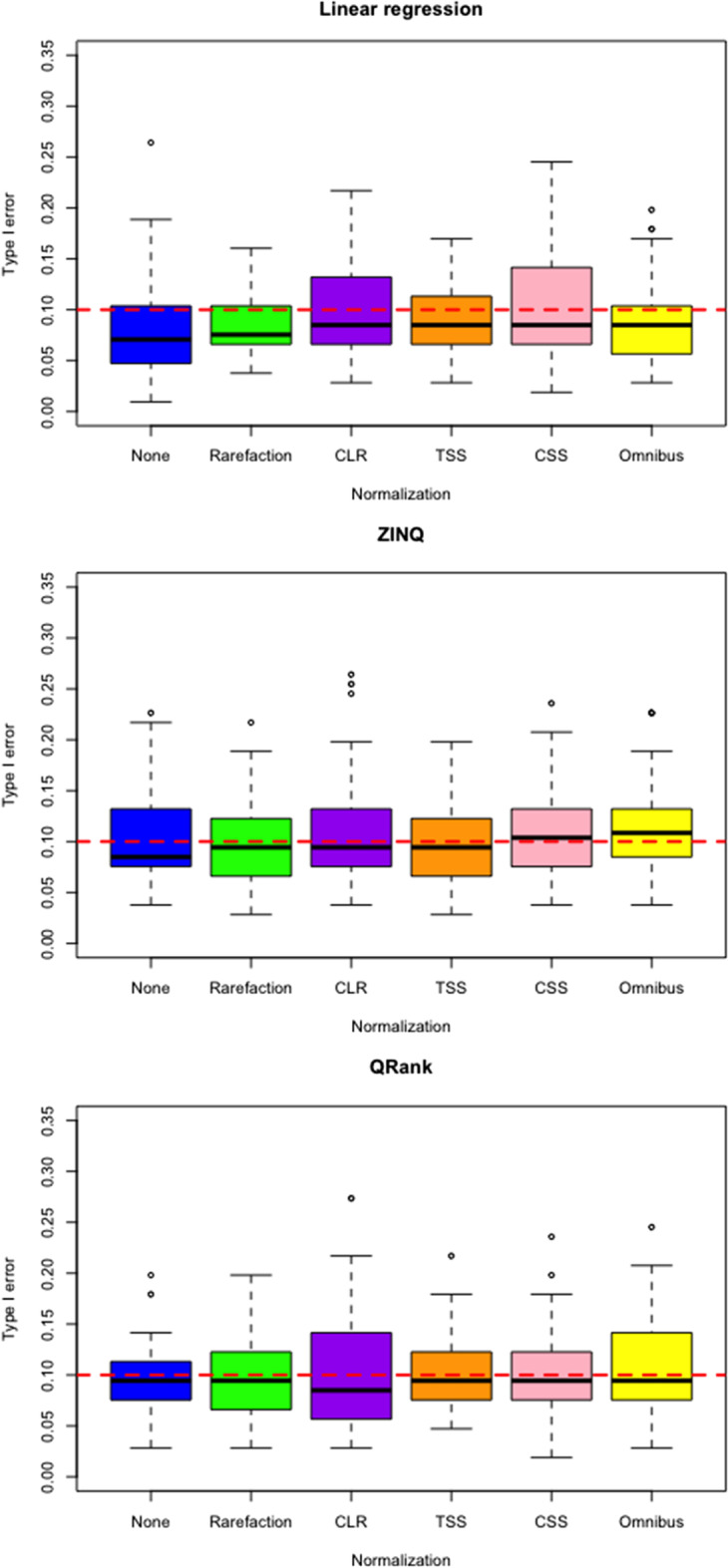
Boxplots of type I error rate under different normalization strategies without adjustment for covariates

**Fig. 3:**
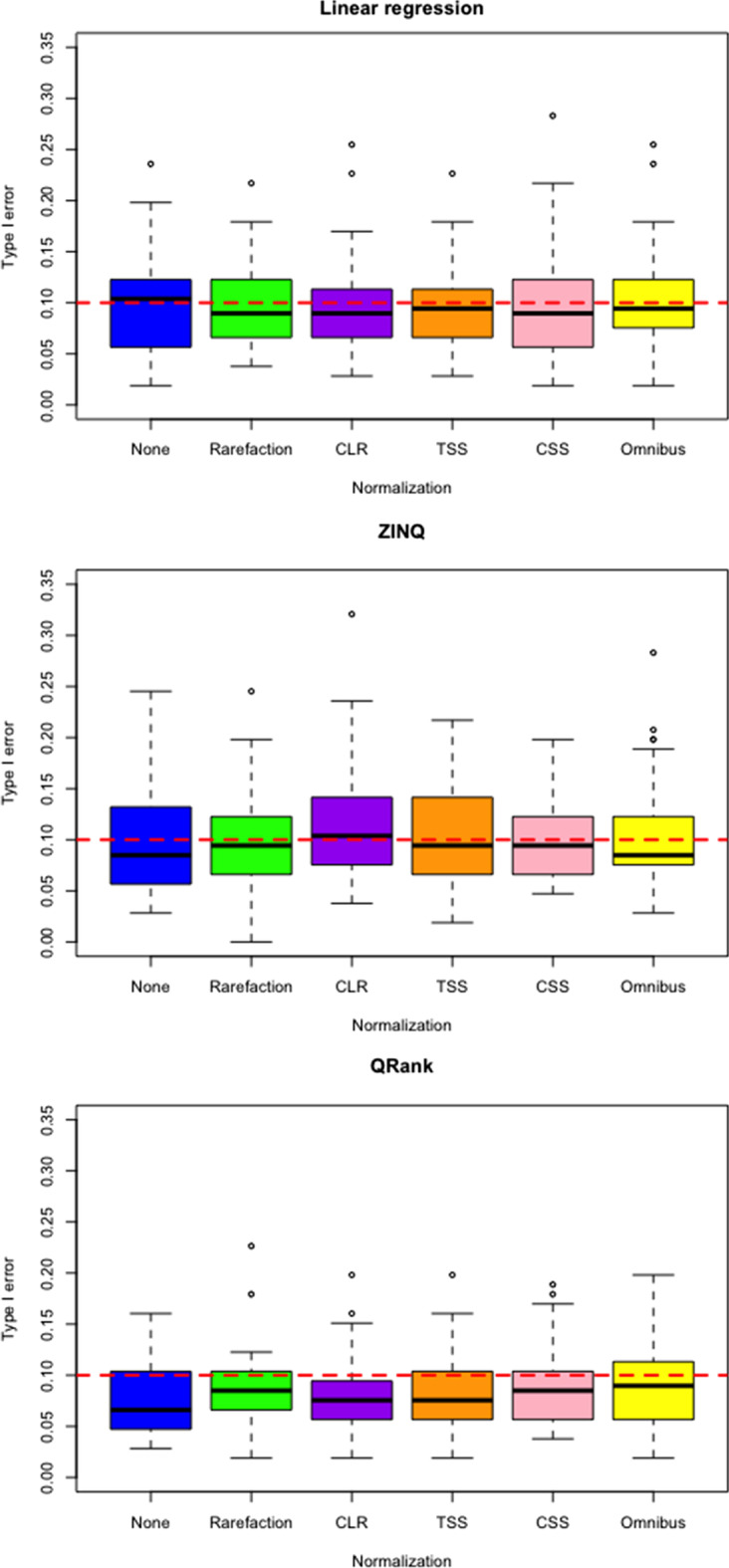
Boxplots of type I error rate under different normalization strategies with adjustment for covariates

We also assess type I error control based on permuted HIV datasets to check the validity of the omnibus test. For each normalization strategy, covariates as well as the sexual orientation are jointly permuted over the whole samples to create a permuted OTU table. This removes the association between the mucosal microbioal abundance and the sexual orientation, and taxa with small *p* values are considered false positive signals. We evaluate type I error control by the proportion of taxa with *p* values less than 0.1. We repeat this procedure 50 times and the results are presented via boxplots (Figs. 2 and 3). Compared to other normalization strategies, we see that the omnibus approach consistently controls the type I error rate well.

## Discussion

We propose the omnibus approach to accommodate multiple potential normalization strategies. Essentially, each choice of normalization inherently assumes a different model for the relationship between taxa and variables of interest, with the optimal choice being unknown (and potentially differing across taxa). By using the omnibus approach, we can avoid the problem of choosing the optimal normalization strategy and instead test across a range of different normalization approaches. Numerical experiments and the real data application demonstrate that the omnibus test not only avoids the possibility of choosing the worst-performing normalization method but also exhibits power nearly as high as the power of the best normalization strategy.

Other factors, such as sequencing methods, amplicon bias, and target gene copy number could impact the differential abundance analysis results. However, they generate bias in microbiome sequencing, making measured relative abundances systematically different from their underlying truth. In this case, bias-resistant modeling or bias-insensitive analytical methods are needed. However, the impact of bias is usually not handled via normalization approaches, and thus goes beyond the scope of this paper. This paper mainly handles the differences caused by differential library sizes that affect all taxa more or less evenly. The focus of this paper is to study whether the omnibus approach helps bypass the normalization issue in microbiome differential abundance testing.

Certain transformations, in principle, can reduce the impact of compositional effects. However, without extrinsic information, there ultimately remains a particular denominator for normalization. Thus, our approach does not seek to overcome the issue, but rather we note that the statistical analysis is “valid” under any choice of normalization and compositionality is an issue of subsequent interpretation. That is, interpretation of significant findings is affected by the compositionality of the data and potentially by the normalization approaches considered, but fully assessing the impact of compositionality on the omnibus test lies outside the scope of the present work.

In this paper, we focus on the linear regression, ZINQ, QRank, and KRV. We acknowledge that a wide range of alternative methods for differential abundance analysis could also be used [42–44]; however, many of these methods are specialized in particular normalizations. For example, a metagenomeSeq method proposed by [43] internally implements CSS normalization and a DESeq2 method proposed by [45] implements relative log expression (RLE) normalization. More importantly, many of these approaches often fail to control the type I error [26, 46, 47] and are, therefore, statistically invalid. In contrast, the simple linear regression, ZINQ, QRank, and KRV have been shown to consistently protect the type I error and do not depend on the particular choice of normalization. We could also combine results across different association testing methods, as well as normalizations, but given their lack of statistical validity, aggregating invalid results just results in further false positives.

In addition, we consider four normalization strategies, including the rarefaction, TSS, CSS, and CLR transformations. Other normalization methods are also commonly used, such as an additive log-ratio transformation (ALR) or an isometric log-ratio transformation (ILR) [48]. However, ALR is computed with respect to the last element of variables, so it heavily depends on this element and its noise. Though ILR has good theoretical properties, it is difficult to interpret the result since transformed variables are intricate mixtures of the original variables. To fairly examine the performance of our approach, we choose four normalization methods that are simpler and more flexible to use.

## Supplementary Information

**Additional file 1** Figure on histograms of some simulated abundances vs. true abundances.

## Data Availability

Data used in this article is available from the CARDIA Study Data Coordinating Center at the University of Alabama at Birmingham. The process for obtaining data through CARDIA is outlined at https://www.cardia.dopm.uab.edu/publications-2/publications-documents. The HIV dataset and R scripts used during the current study are available from the corresponding author upon reasonable request. The main function code for the omnibus approach with an example code is available at https://github.com/hoseungs/Omnibus_normalization.

## Abbreviations

CCT: Cauchy combination test
TSS: Total sum scaling
CSS: Cumulative sum scaling
CLR: Center log ratio transformation
CARDIA: The coronary artery risk development in young adults study dataset
HBP: High blood pressure
EDF: Empirical distribution functions
ART: Antiretroviral therapy
MSM: Men who have sex with men
FDR: False discovery rate
RLE: Relative log expressions

## Footnotes

1 https://www.metagenomics.wiki/tools/16s/norm/css
