# Supplementary material for: Accommodating multiple potential normalizations in microbiome associations studies
Source: BMC Bioinformatics. 2023 Jan 19;24:22. doi: 10.1186/s12859-023-05147-w (PMC9850542; doi:10.1186/s12859-023-05147-w)
Supplement: Supplementary file 1 — Additional file 1 Figure on histograms of some simulated abundances vs. true abundances. [file 12859_2023_5147_MOESM1_ESM.pdf]

Additional file 1 – Supplementary information

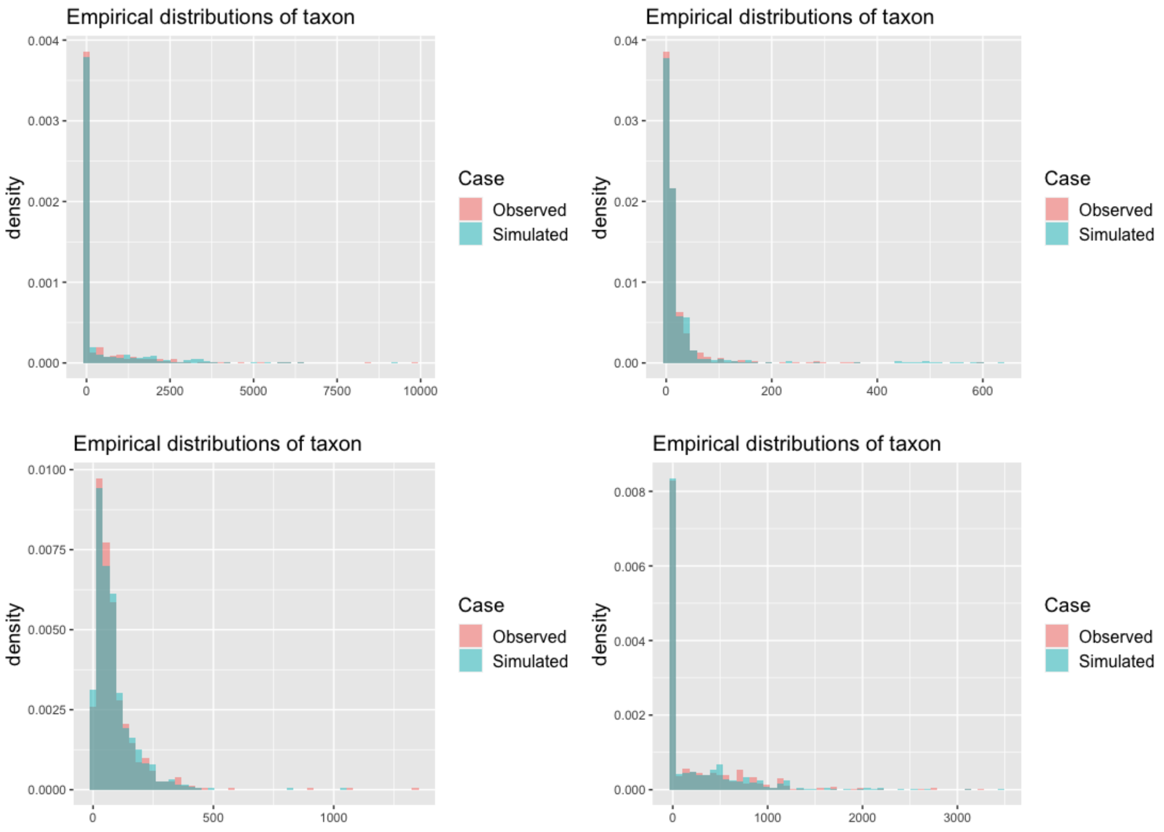

Figure S1: Histograms of some simulated abundances vs. true abundances.

For Setting 2, the two-part quantile model preserves the zero-inflation characteristics of the taxa, and also captures every quantile of the positive abundances, preserving the over-dispersion and even other characteristics of the irregular distributions. Any kind of modeling can only approximate the truth as much as it can, while the quantile modeling can pre- serve more details than parametric choices such as models based on the Dirichlet- multinomial distribution. We compare some simulated abundances with the observed abun- dances from the data by using histograms (Additional file 1: Figure S1). We see that the simulated abundances based on the quantile model is similar to the true abundances.
